# Supervised team management, with or without structured psychotherapy, in heavy users of a mental health service with borderline personality disorder: a two-year follow-up preliminary randomized study

**DOI:** 10.1186/1471-244X-11-181

**Published:** 2011-11-21

**Authors:** Federico Amianto, Andrea Ferrero, Andrea Pierò, Elisabetta Cairo, Giuseppe Rocca, Barbara Simonelli, Simona Fassina, Giovanni Abbate-Daga, Secondo Fassino

**Affiliations:** 1 Neurosciences Department, Psychiatry Section, University of Torino, Via Cherasco 11, Turin, Italy; 2 Psychotherapy Unit, Mental Health Department, Health District TO-4, Via Don Paviolo 5, Settimo T.se, Turin, Italy; 3 SAIGA Institute of Research, Via Principe Amedeo 16, Turin, Italy; 4 Centre Hospitalier Alpes-Isère, Grenoble, 38000, France

## Abstract

**Background:**

Individuals affected by severe Borderline Personality Disorder (BPD) are often heavy users of Mental Health Services (MHS). Short-term treatments currently used in BPD therapy are useful to target disruptive behaviors but they are less effective in reducing heavy MHS use. Therefore, alternative short-term treatments, less complex than long-term psychodynamic psychotherapies but specifically oriented to BPD core problems, need to be developed to reduce MHS overuse. This study aimed to evaluate the efficacy of adding Sequential Brief Adlerian Psychodynamic Psychotherapy (SB-APP) to Supervised Team Management (STM) in BPD treatment compared to STM alone in a naturalistic group of heavy MHS users with BPD. Effectiveness was evaluated 6 times along a two-year follow-up.

**Methods:**

Thirty-five outpatients who met inclusion criteria were randomly assigned to two treatment groups (STM = 17; SB-APP = 18) and then compared. Clinical Global Impression (CGI) and CGI-modified (CGI-M) for BPD, Global Assessment of Functioning (GAF), State-Trait Anger Expression Inventory (STAXI), and Symptom Checklist-90 Revised (SCL-90-R) were administered at T1, T3, T6, T12, T18 and T24. At T12 the Working Alliance Inventory-Short Form (WAI-S) was also completed. At the one-year follow-up, SB-APP group did not receive any additional individual psychological support. MHS team was specifically trained in BPD treatment and had regular supervisions.

**Results:**

All patients improved on CGI, GAF, and STAXI scores after 6 and 12 months, independently of treatment received. SB-APP group showed better outcome on impulsivity, suicide attempts, chronic feelings of emptiness, and disturbed relationships. We found a good stabilization at the one year follow-up, even after the interruption of brief psychotherapy in the SB-APP group.

**Conclusions:**

Although STM for BPD applied to heavy MHS users was effective in reducing symptoms and improving their global functioning, adding a time-limited and focused psychotherapy was found to achieve a better outcome. In particular, focusing treatment on patients' personality with a specific psychotherapeutic approach (i.e. SB-APP) seemed to be more effective than STM alone.

**Trial Registration:**

ClinicalTrials.gov: NCT1356069

## Background

Borderline Personality Disorder (BPD) is a severe disorder with substantial social cost [1]. Individuals affected by severe BPD are often heavy users of psychiatric and medical services [2], entailing high costs for Mental Health Services (MHS). A previous study conducted by Ferrero and Coworkers [3] highlighted that, during a three-year period of data collection in an Italian MHS, about 10% of patients, which were all heavy users of the service, used nearly the 50% of the available resources. About 50% of these heavy users were affected by BPD.

Clinical experience, supported by a recent systematic review of literature [4], shows that both BPD severity of symptoms and social impairment are not adequately improved by medications. Scientific evidence demonstrates that, to interrupt this pattern of high use, the lack of effective drug treatments should be balanced by the addition of psychotherapy within the available treatment options [2]. Other studies sustain that the management of heavy MHS users could be improved by training MHS team about psychological core dimensions of BPD [5].

Currently, both cognitive-behavioral and psychodynamic psychotherapies for BPD seem effective to reduce psychopathological severity [6]. It has been also suggested that long-term treatments could be useful to avoid drop-out in patients with attachment disturbances [7]. Nevertheless, these approaches are often unavailable due to the lack of resources and they do not resemble to treatment as usual [8]. In current practice, MHS users affected by BPD often do not undergo psychotherapy and moreover the staff is not specifically trained in BPD treatment [3].

On the other hand, short-term treatments which are currently effective for BPD individuals as Dialectical Behavior Therapy (DBT) [9] are useful to target some specific disruptive behaviors of severe BPD, but they are less effective in reducing heavy MHS use, probably because of affectivity core features, such as intolerance of aloneness and conflicts on dependence [10].

Another issue, related to the affective core of BPD is the tendency to "pushing the limits" in building therapeutic alliance. This is not necessarily related to self-damaging or disrupting behaviors but it may produce a high rate of MHS use and difficulties in clinical management [11]. New kinds of treatment, focused on the affective "core" of BPD patients, are thus needed to reduce MHS overuse.

Short-term treatments, less complex than long-term psychodynamic psychotherapies but more tailored to the extensive problems of BPD individuals, need to be developed in order to reduce their heavy MHS use [6,8]. Furthermore, MHS team need to receive specific training, supervision and support in these models of psychotherapy [5] to promote a clinical coherent view [12].

As highlighted by Kerr [5], the effects of these therapeutic approaches on severe BPD population still received limited investigations. Moreover, since the adjunct of a time-limited psychodynamic psychotherapy to Supervised Team Management (STD), is more complex than the addiction of a specific training alone to MHS staff, it is necessary to prove that time-limited psychodynamic psychotherapy is really cost-effective for BPD patients' outcome.

The aim of this study was to compare the effectiveness of STM comprehending specific staff training and supervision to the same treatment with the addition of a time-limited weekly psychodynamic psychotherapy (Sequential Brief Adlerian Psychodynamic Psychotherapy, SB-APP) on randomized BPD patients who meet the criteria for heavy and long-term users of MHS referring to a specific mental health outpatient service.

Moreover, we aimed to show that SB-APP is useful to achieve a better outcome in general functioning of BPD patients, who are high and long-term users of MHS, with respect to STM. In order to identify some peculiar therapeutic elements [13], we hypothesized that these two interventions could be addressed to different core psychopathological features of BPD [14,15]: supportive as usual interventions are focused on individuals' impairment while SB-APP is carefully focused on personality functioning.

## Methods

### Patients

We started recruiting on January 1^st^, 2004, until January 1^st^, 2008. We enrolled 81 users of the outpatient service of the Mental Health Center of Chivasso (Turin), Italy, who had been treated and clinically managed at least one year. Inclusion criteria were: (a) diagnosis of BPD according to DSM-IV-TR criteria; (b) age ranging between 20 and 50 years; (c) heavy use of MHS throughout the prior year; (d) absence of an acute comorbid Axis I disorder requiring hospitalization; (e) no current Substance Dependence Disorder; (f) no Mental Retardation; (g) no previous psychotherapy interventions; and (h) valid informed consent. Diagnostic assessments for Axes I and II disorders were conducted at baseline by two trained psychiatrists, using the Structured Clinical Interview for DSM-IV [16,17].

Heavy and long-term use was defined as follows, according to previous literature [3]: a) more than six interventions in emergency departments in the prior year; b) more than 52 outpatient visits in the prior year, and c) more than 12 unscheduled outpatient interventions by psychiatrists in the prior year. Patient could be recruited in the study if at least only one criteria was met.

**Figure 1:**
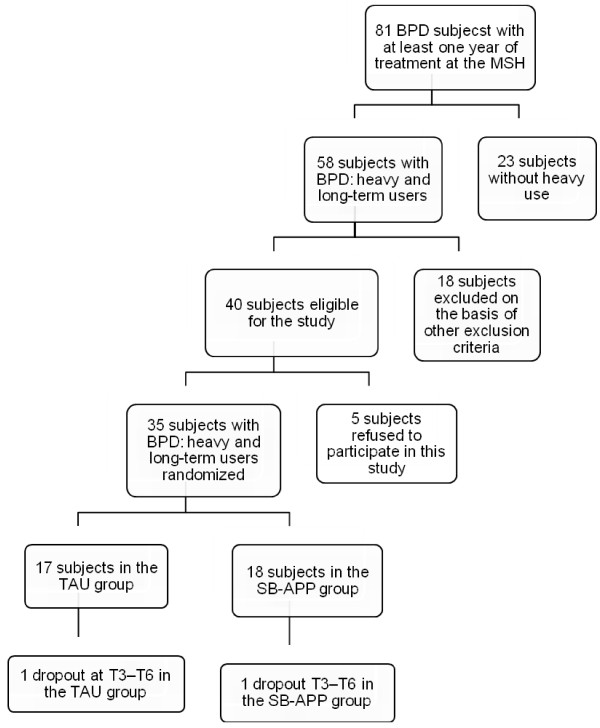
**Patients' selection and dropout**. The figure displays the flow-chart of patients' selection and dropout during the course of the recruitment, treatment and follow-up.

Figure 1 shows the flowchart of recruitment. Among the whole sample of BPD patients, 23 did not meet criteria for heavy and long-term use of MHS. Eighteen BPD patients with heavy users features were also excluded due to several factors: age (n = 2); acute Axis I disorder requiring hospitalization (n = 8); comorbid Mental Retardation (n = 2) or Substance Dependence Disorder (n = 6). Five patients who met the inclusion criteria refused to participate in the study or to provide a valid informed consent and five patients refused to fill in the self-administered questionnaires. Therefore, the final sample consisted of 35 heavy users of MHS with a diagnosis of BPD.

### Procedures

All procedures were approved by the Institutional Ethic Committee of Chivasso Mental Health District and were conduced according to the 1995 Declaration of Helsinki as revised in Edinburgh in 2000.

Patients, after providing their informed consent, were randomly assigned to SB-APP in addition to Supervised Team Management (STM; n = 18) or to STM alone (n = 17) groups. The random allocation was generated using a random number table and was carried out by one author (BR), who was not involved in patients' clinical management. Patients were enrolled by a psychiatrist of the MHS who communicated the generalities of the participants who signed the informed consent to BR and received from BR the group assignment. The treatment allocation was assured by both psychiatrist and psychotherapist. Then the psychiatrist explained to participants that all treatments considered in this study were supposed to be effective. The team was informed by the psychiatrist of patients' allocation to each branch of the study; each participant was assigned to a psychotherapist who knew what treatment the patient was undergoing. The psychotherapist structured the sessions with the patient accordingly to treatment assignment.

Treatments of each branch were conduced simultaneously and both started the week after the enrollment in the study.

To measure treatment efficacy four assessments were set for both self-administered and hetero-administered scales: at intake (T0), after three (T3), six (T6), and 12 months (T12). Two further time points at 18 months (T18) and 24 months (T24) from enrollment were set for hetero-evaluation only.

STM group received the usual treatment for 12 months. STM treatment also included sessions of psychological support focused on patients' social impairment, which were not structured in a time-limited psychotherapy treatment. SB-APP group received the usual treatment plus SB-APP (40 weekly sessions) for 10 or 11 months. At the end of the first year (T12), STM group continued with the as-usual management with supportive weekly sessions whilst the SB-APP group was carried on with psychiatric, nurse and educational management without any individual psychological support. The number of sessions performed by the two groups in the first year (T0-T12) was planned to be comparable, reducing the bias about the number of session.

All psychotherapists were licensed graduate psychologists and all of them had a personal analytic training and a specific training in SB-APP. Psychotherapists were not electively assigned to one group or another but could indifferently perform single supportive sessions with certain patients and a structured cycle of SB-APP with others. In this way both influence of personal skills and attitudes of the therapists on the outcome of the study could be reduced.

### Supervised team management (STM)

STM included: (a) medications, (b) unstructured psychological support focused on socio-relational impairment, (c) rehabilitative interventions, and (d) MHS training. Medications were administered according to APA guidelines for BPD [18]. Three classes of drugs were used: antidepressants (selective serotonin reuptake inhibitors [SSRI] or serotonin-noradrenalin reuptake inhibitors SNRI/noradrenergic and specific serotonergic antidepressants [NaSSA]s), mood stabilizers (valproate, lamotrigine), and atypical antipsychotics (olanzapine, aripiprazole, risperidone, quetiapine). Drug treatment was prescribed during the first or the second visit and modified if necessary during follow-up by the clinical manager. Side effects were monitored when clinically significant. Psychological support was provided by psychotherapists while rehabilitative interventions by nurses, educators, or psychologists.

Since the beginning of the study, the multidisciplinary MHS Team of Chivasso in Italy (psychiatrists, psychologists, nurses and educators) was trained in BPD treatment [12]. They were involved in: 1) preliminary brief educational program concerning etiology, symptoms and care of BPD; 2) regular supervisions, in order to promote a coherent treatment planning; 3) biweekly case supervision sessions (lasting 90 minutes), and monthly case discussions (lasting 60 minutes) to share professional and emotional loads. The conductor was a psychodynamic-oriented psychiatrist, trained in group dynamics.

No other changes were made to the as-usual approach to BPD patients recruited in the study. Thus, the team was free to manage therapy, sessions and other procedures without any limitation and on the basis of clinical needs. The only exception was the assignment of randomly selected participants to a cycle of psychotherapy (SB-APP).

### Sequential Brief-Adlerian Psychodynamic Psychotherapy (SB-APP)

SB-APP, derived from Brief-Adlerian Psychodynamic Psychotherapy (B-APP) [19,20], is a time-limited (40 weekly sessions) psychodynamic psychotherapy based on Alfred Adler's theory and delivered in sequential and repeatable modules [20].

SB-APP is focused specifically on four Personality Functioning Levels (PFL). These are assessed by the therapists on the basis of symptoms, quality of interpersonal relationships, overall social behaviors, cognitive and emotional patterns, and defense mechanisms [21]. At PFL 1, SB-APP is focused on preventing disruptive acting-out by providing reality testing by strengthening self-reflective functions and identity. At PFL 2, the approach is focused on increasing empathy through validating thoughts and emotions and decreasing the sense of emptiness, egocentrism, and dependence. At PFL 3, therapy aims at reducing idealization and increasing continuity and adaptation. At PFL 4, it attempts to develop increased tolerance for ambivalence, help patients overcome conflicts, enhance autonomy, and increase positive attitudes toward the project [20].

SB-APP is devoted to building a favorable working alliance. Practitioners conceptualize interventions along a continuum and do not make a sharp distinction between encouraging the patient to search for solutions and providing them (Encouraging Position Axis, EP-AX) or between explorative (i.e., interpretation and confrontation) or validating (i.e., empathic validation) techniques (Technical Instruments Axis, TI-AX). Analogously, psychological functioning is placed along a continuum for the purpose of treatment planning. Intensive psychotherapies are mainly characterized by the elaboration of personal experiences by the patient; while supportive ones are mainly characterized by the integration of the therapist's contributions (Intensive Supportive Axis, IS-AX). Nevertheless, the therapist selects each "technical instrument" to serve either a conservative (respecting and strengthening the patient's defensive structure) or mutative (provoking a change in the patient's defense mechanisms) purpose (Mutative Conservative Axis, MC-AX) on the basis of the patient's personality organization [20,22].

In order to preserve compliance to treatment, all psychotherapists underwent biweekly supervisions in addition to monthly case discussions. The supervisor (AF, one of the theorists and teaching psychotherapists of SB-APP) ascertained that all therapists could properly identify and distinguish PLF 1, 2, 3, 4 accordingly to their aforementioned definition. Moreover, he monitored their adherence to SB-APP technique and its application accordingly to participants' PLF.

### Measures

Participants were asked to complete several questionnaires and rating scales were administered by two independent raters at each follow-up: at T0 it was only administered a dimensional personality inventory (Temperament and Character Inventory - TCI); at T0, T3, T6, T12 some self-rated psychopathological measures (Symptom Checklist-90 - SCL-90; State-Trait Anger Expression Inventory - STAXI); at T0, T3, T6, T12, T18, T24 hetero-administered rating scales (Clinical Global Impression - CGI, Global Assessment of Functioning - GAF, Clinical Global Impression Modified - CGI-M).

The Working Alliance Inventory-Short Form (WAI-S) was filled in by participants only at T12, to evaluate the different degree of therapeutic alliance between SB-APP and STM groups after treatment. The raters were trained in the use of this rating scale, to ensure good internal consistency and inter-rater reliability, and they were blind with regard to the group assignment of patients.

*Global Assessment of Functioning (GAF) *evaluates the level of social and occupational functioning of the individual.

*Clinical Global Impression (CGI) *is a one-item assessment tool administered by clinicians to evaluate severity of illness with a score ranging from 0 (non-assessed) to 7 (extreme severity).

*Clinical Global Impression-Modified (CGI-M) *uses the same scoring system of the CGI to assess the severity of each of nine items included in the DSM-IV diagnostic criteria for BPD [23]: 1) fear of abandonment; 2) disturbed relationships; 3) identity distortion; 4) impulsivity; 5) suicidality and self-damaging acts; 6) affective instability; 7) chronic feelings of emptiness; 8) anger reactions; and 9) dissociative symptoms.

*Symptom Checklist-90 Revised (SCL-90-R) *[24] is a self-report tool, used to identify psychopathological distress, consisting of nine psychopathological dimensions: I. Somatization; II. Obsessive-Compulsiveness; III. Interpersonal Sensitivity; IV. Depression; V. Anxiety; VI. Hostility; VII. Phobic Anxiety; VIII. Paranoid Ideation; and IX. Psychoticism. In consideration of the high number of scales of this study and of the heterogeneity of BPD symptomatology, only SCL-90-R total score, without subscales, was considered as reliable outcome measure. It has been used as raw score in statistical analyses.

*State-Trait Anger Expression Inventory (STAXI) *[25] is a 44-item self-report questionnaire that measures the experience and expression of anger. It consists of 44 items divided into six scales and two subscales. The Trait-Anger (T-ANG) scale includes two subscales: T-Anger/T, which measures angry temperament, and T-Anger/R, which measures reaction to criticism. The Anger Expression-In (AX-IN) scale measures anger suppression, the Anger Expression-Out (AX-OUT) scale measures the anger expression toward other people or objects, and the Anger Expression-Control (AX-CON) scale measures the frequency of attempts to control anger. Finally, the AX/EX scale provides a general index of the expression of anger.

*The Working Alliance Inventory-Short Form, client version (WAI-S) *measures the working alliance in therapeutic settings [26]. The WAI-S is a 12-item self-report questionnaire consisting of three subscales: 1) how closely the client and therapist agree about and are mutually engaged in pursuit of the goals of treatment; 2) how closely the client and therapist agree about how to reach the treatments goals; and 3) the degree of mutual trust, acceptance, and confidence between the client and therapist. The composite score is used as a global measurement of the working alliance. Respondents are asked to rate each statement on a 7-point Likert scale ranging from 1 (never) to 7 (always). The total score ranges from 12 to 84, with higher scores indicating a stronger working alliance.

Among clinical data, time from the first contact and number of hospitalization days during the year before recruitment (Table 1) were compared at T0 to ascertain the comparability of these two randomized samples. Days of hospitalization and self-harming episodes were registered at T0 (Table 1), T12 and T24, referring to the prior year.

**Table 1:** T0 Comparison of STM and SB-APP groups.

	STM(n = 17)	SB-APP(n = 18)	*t*-test	*p *<
Age	40.1 ± 10.4	39.2 ± 8.6	.26	.797
Education	9.11 ± 1.8	9.38 ± 2.0	-.41	.679
Time from the first contact	7.3 ± 6.1	7.9 ± 6.3	-.31	.757
Inpatient treatment (days)*	21.1 ± 58.1	31.2 ± 66.6	-.48	.634
Self harming incidents	2.3 ± 3.1	3.1 ± 2.5	.71	.405
SCL90-R Total	121.9 ± 86.6	160.1 ± 69.2	-1.44	.158
CGI item 1 T0	3.8 ± .8	4.3 ± .9	-1.68	.102
GAF T0	57.2 ± 9.6	60.2 ± 9.1	-.94	.352
AX-EX T0	31.4 ± 13.2	33.0 ± 11.8	-.37	.710

	**STM**	**SB-APP**	***chi*-square**	***p *<**

Females	9 (53%)	8 (44%)	.253	.615
Males	8 (47%)	10 (56%)		

*in the year before recruitment in the study

SCL-90-R Tot = Symptom Checklist 90 - Revised, Total score; CGI Item 1 = Clinical Global Impression; GAF = Global Assessment of Functioning; AX-EX = anger expression (STAXI). STM = Supervised Team Management group; SB-APP = Sequential Brief Adlerian Psychodynamic Psychotherapy group

*df *= 1

The first criterion of MHS high use was considered as main clinical outcome: i.e. requiring more than six emergency interventions in the prior year.

### Statistical Analyses

All data analyses were performed using the Statistical Package for the Social Sciences (SPSS, 16.0). Comparison between STM and SB-APP groups was performed at intake (T0) to assess randomization effects using a *t-*test for continuous and a *chi*-square test for categorical variables (Table 1). The questionnaire (SCL-90-R, STAXI) and rating scale (CGI, CGI-M items 1-9, GAF) scores obtained by the 35 patients at all assessments (T0, T3, T6, and T12) were compared using a general linear model ANOVA (GLM - ANOVA) for repeated measures. The effects of time and time*group were also analyzed (Tables 2 and 3).

**Table 2:** T0, T6, T12, T24 comparisons during the first year of the study: self-administered questionnaires.

VARIABLE	Times of observation				GLM*	*p *<	GLM**	*p *<
	T0 (17 vs 18)	T3 (17 vs 18)	T6 (16 vs 17)	T12 (16 vs 17)				
SCL-90-R tot								
STM	130.9 ± 85.2	102.1 ± 71.7	93.3 ± 62.5	93.5 ± 67.2	7.78	**.000**°	.55	.645
SB-APP	156.6 ± 70.0	137.9 ± 74.4	115.6 ± 72.0	103.1 ± 83.4				
S-ANG								
STM	17.1 ± 9.0	15.6 ± 7.2	14.2 ± 6.8	13.8 ± 6.5	.40	.747	.87	.458
SB-APP	17.1 ± 8.3	17.7 ± 7.4	16.6 ± 10.4	19.0 ± 12.5				
T-ANG								
STM	19.9 ± 6.8	19.0 ± 7.3	17.4 ± 7.7	18.8 ± 6.3	.33	.569	.14	.709
SB-APP	22.5 ± 7.9	22.5 ± 6.8	21.9 ± 8.8	22.4 ± 8.8				
AX-IN								
STM	19.5 ± 4.4	18.8 ± 6.4	18.6 ± 5.0	18.7 ± 5.6	1.24	.298	.35	.785
SB-APP	20.5 ± 4.2	20.2 ± 5.5	18.7 ± 2.9	18.8 ± 3.1				
AX-OUT								
STM	16.0 ± 6.2	16.9 ± 5.3	15.7 ± 4.3	15.3 ± 4.4	1.19	.317	.33	.803
SB-APP	17.0 ± 4.9	16.4 ± 2.9	16.1 ± 3.7	15.4 ± 4.5				
AX-CON								
STM	20.9 ± 5.9	19.7 ± 5.5	22.2 ± 6.6	23.8 ± 6.6	6.44	**.001°**	1.33	.269
SB-APP	19.5 ± 6.0	22.4 ± 3.6	23.4 ± 5.8	25.1 ± 4.2				
AX-EX								
STM	29.3 ± 13.2	31.9 ± 14.1	28.8 ± 12.7	31.3 ± 9.5	1.01	.391	1.41	.245
SB-APP	33.8 ± 10.7	32.3 ± 9.1	29.8 ± 11.1	27.9 ± 10.4				

SCL-90-R Tot = Symptom Checklist 90 - Revised, Total score; S-ANG = state-anger; T-ANG = trait anger; AX-IN = anger expressed inwards; AX-OUT = anger expressed outwards; AX-CON = anger control; AX-EX = anger expression; * = changes within individuals; ** = differences between treatment groups. **° ***post-hoc***: **SCL-90-R Tot = T6, T12 < T0; AX-CON = T6 > T0.

STM = Supervised Team Management group; SB-APP = Sequential Brief Adlerian Psychodynamic Psychotherapy group

*df *= 3

**Table 3:** T0, T6, T12, T24 comparisons during the first year of the study: rating scales.

VARIABLE	Times of observation				GLM*	*p *<	GLM**	*p *<
	T0 (17 vs 18)	T3 (17 vs 18)	T6 (16 vs 17)	T12 (16 vs 17)				
CGI Item 1								
STM	3.8 ± .8	3.2 ± .8	3.3 ± .7	3.3 ± 1.0	8.47	**.000°**	1.33	.258
SB-APP	4.3 ± 1.0	3.7 ± 1.1	3.6 ± 1.1	3.3 ± 1.1				
GAF								
STM	57.4 ± 9.9	64.1 ± 7.8	65.9 ± 11.6	65.9 ± 11.2	11.61	**.000°**	2.31	.081
SB-APP	60.2 ± 9.1	62.5 ± 12.2	62.3 ± 12.4	66.4 ± 13.0				
CGI-M 1								
STM	3.4 ± 1.4	3.1 ± 1.3	2.9 ± 1.3	2.4 ± 1.1	23.27	**.000°**	2.55	.060
SB-APP	4.2 ± 1.2	3.5 ± 1.6	2.8 ± 1.2	2.6 ± 1.1				
CGI-M 2								
STM	3.1 ± 1.4	3.0 ± 1.5	2.9 ± 1.2	2.4 ± 1.1	17.68	**.000°**	2.88	**.040°°**
SB-APP	4.3 ± 1.5	3.8 ± 1.3	3.2 ± 1.2	2.8 ± 1.3				
CGI-M 3								
STM	3.2 ± 1.5	2.7 ± 1.1	2.4 ± 1.1	2.2 ± .1	19.69	**.000°**	.24	.869
SB-APP	4.4 ± 1.0	3.9 ± 1.1	3.3 ± 1.0	3.2 ± 1.2				
CGI-M 4								
STM	2.6 ± 1.3	2.5 ± .7	2.2 ± 1.0	1.8 ± 1.1	18.14	**.000°**	3.24	**.025°°**
SB-APP	3.5 ± 1.4	2.6 ± 1.3	2.4 ± 1.1	2.0 ± 1.0				
CGI-M 5								
STM	2.1 ± 1.5	1.7 ± 1.0	1.6 ± .7	1.4 ± .7	25.03	**.000°**	6.09	**.019°°**
SB-APP	3.5 ± 1.9	2.9 ± 1.9	1.8 ± .8	1.5 ± .8				
CGI-M 6								
STM	2.8 ± 1.3	2.7 ± 1.0	2.4 ± .9	2.2 ± .9	9.61	**.000°**	1.42	.241
SB-APP	4.3 ± 1.4	3.8 ± 1.5	3.3 ± 1.4	2.9 ± 1.4				
CGI-M 7								
STM	3.2 ± 1.3	2.9 ± 1.3	2.8 ± 1.2	2.6 ± 1.1	19.23	**.000°**	2.50	**.009°°**
SB-APP	4.2 ± 1.2	3.7 ± 1.4	3.4 ± 1.2	2.8 ± .8				
CGI-M 8								
STM	3.4 ± 1.2	3.3 ± 1.3	2.9 ± 1.1	2.6 ± 1.1	12.32	**.000°**	.64	.592
SB-APP	3.6 ± 1.5	3.1 ± 1.3	2.9 ± 1.2	2.6 ± 1.4				
CGI-M 9								
STM	2.4 ± 1.1	2.2 ± 1.1	1.6 ± .9	1.6 ± .9	10.77	**.000°**	1.87	-139
SB-APP	2.9 ± 1.9	2.7 ± 1.8	2.7 ± 1.8	2.3 ± 1.5				

STM = Supervised Team Management group; SB-APP = Sequential Brief Adlerian Psychodynamic Psychotherapy group; CGI Item 1: Clinical Global Impression; CGI-M: CGI modified for BPD subjects. Items: 1) fear of abandonment; 2) disturbed relationships; 3) identity distortion; 4) impulsivity; 5) suicidality; 6) affective instability; 7) chronic feelings of emptiness; 8) anger reactions; 9) dissociative symptoms. * = changes within individuals; ** = differences between treatment groups; *post-hoc ***° **= T12 < T6 < T3, T1; **°° = **SB-APP group showed a greater tendency toward improvement. *df *= 3

Using GLM - ANOVA for repeated measures the rating scales (CGI, CGI-M items 1-9, GAF) were compared at the three follow-up observations (T12, T18, and T24). The effects of time and time*group were also analyzed (Tables 3 and 4).

**Table 4:** T0, T6, T12, T24 comparisons during the second year of the study: rating scales.

VARIABLE	Times of observation			GLM*	*p *<	GLM**	*p *<
	T12 (16 vs 17)	T18 (16 vs 17)	T24 (16 vs 17)				
CGI Item 1							
STM	3.3 ± 1.0	3.2 ± .9	3.1 ± 1.0	.36	.696	1.79	.174
SB-APP	3.3 ± 1.1	3.4 ± 1.0	3.4 ± .9				
GAF							
STM	65.9 ± 11.2	64.7 ± 14.0	65.6 ± 13.4	.53	.589	.01	.987
SB-APP	66.4 ± 13.0	65.6 ± 13.0	66.2 ± 11.9				
CGI-M 1							
STM	2.4 ± 1.1	2.4 ± 1.1	2.3 ± 1.0	.19	.827	.41	.661
SB-APP	2.6 ± 1.1	2.6 ± 1.0	2.7 ± 1.0				
CGI-M 2							
STM	2.4 ± 1.1	2.1 ± 1.1	2.2 ± 1.1	1.46	.238	.24	.784
SB-APP	2.8 ± 1.3	2.7 ± 1.2	2.8 ± 1.2				
CGI-M 3							
STM	2.2 ± 1.0	2.2 ± 1.1	2.0 ± 1.2	.16	.855	1.02	.365
SB-APP	3.2 ± 1.2	3.3 ± 1.2	3.4 ± 1.3				
CGI-M 4							
STM	1.7 ± 1.1	1.9 ± 1.0	2.3 ± .5	1.85	.166	2.17	.122
SB-APP	2.1 ± .9	2.2 ± .7	2.1 ± .6				
CGI-M 5							
STM	1.4 ± .7	1.5 ± .8	1.4 ± .6	.58	.563	.30	.739
SB-APP	1.8 ± 1.0	1.7 ± .9	1.7 ± .9				
CGI-M 6							
STM	2.3 ± .9	2.0 ± 1.0	2.1 ± 1.1	1.03	.362	.04	.958
SB-APP	2.9 ± 1.4	2.7 ± 1.3	2.8 ± 1.5				
CGI-M 7							
STM	2.6 ± 1.1	2.4 ± 1.1	2.2 ± 1.1	2.31	.108	1.32	.274
SB-APP	2.8 ± .8	2.9 ± .8	2.8 ± .9				
CGI-M 8							
STM	2.6 ± 1.1	2.5 ± 1.2	2.4 ± 1.3	1.06	.350	.02	.972
SB-APP	2.6 ± 1.4	2.5 ± 1.3	2.4 ± 1.3				
CGI-M 9							
STM	1.6 ± .9	1.8 ± 1.1	1.4 ± 1.0	1.41	.250	.07	.927
SB-APP	2.3 ± 1.5	2.3 ± 1.7	2.1 ± 1.3				

STM = Supervised Team Management group; SB-APP = Sequential Brief Adlerian Psychodynamic Psychotherapy group; CGI Item 1: Clinical Global Impression; CGI-M: CGI modified for BPD subjects. Items: 1) fear of abandonment; 2) disturbed relationships; 3) identity distortion; 4) impulsivity; 5) suicidality; 6) affective instability; 7) chronic feelings of emptiness; 8) anger reactions; 9) dissociative symptoms. * = changes within individuals; ** = differences between treatment groups. *df *= 2

The number of days of hospitalization and self-harming episodes of BPD participants during the prior year (T0) and at T12 and T24 were compared using the GLM - ANOVA for repeated measures.

*Chi*-square test for categorical variables was used to compare the frequency of high use of MSH in the general sample and in the two subgroups relating it to the GAF scores at T24. The proportion of participants requiring more than six emergency interventions in each treatment group was also compared at each time point and with respect to each group (Table 5).

**Table 5:** T0, T12, T24 comparisons during the second year of the study: clinical outcomes.

VARIABLE	Times of observation			GLM*	*p *<	GLM**	*p *<
	T0 (17 vs 18)	T12 (16 vs 17)	T24 (16 vs 17)				
Inpatient treatment (days)							
STM	21.1 ± 58.1	17.3 ± 53.2	4.8 ± 9.8	4.937	.033	.142	.709
SB-APP	31.2 ± 66.6	26.5 ± 54.7	8.2 ± 19.1				
Self harming incidents							
STM	2.3 ± 3.1	2.2 ± 2.8	.4 ±.8	33.370	.000	1.337	.252
SB-APP	3.1 ± 2.5	1.9 ± 2.0	.3 ±.6				

				*chi*-square*	***p *<**	*chi*-square**	***p *<**

> 6 Emergency Interventions							
STM	12/17(70%)	7/16(40%)	5/16(30%)	5.591	.061	9.764	.135
SB-APP	10/18(61%)	6/17(33%)	3/17(18%)	5.472	.065		

STM = Supervised Team Management group; SB-APP = Sequential Brief Adlerian Psychodynamic Psychotherapy group; * = changes within individuals; ** = differences between treatment groups.

*df *= 2 for GLM and for *chi*-square*; *df *= 6 for *chi*-square**

Finally, GLM - ANOVA was used to compare the total scores for working alliance (WAI-S) and the number of sessions performed at T12 (non-structured or structured with the SB-APP) in STM and SB-APP groups, controlling for sex, time since first contact with the MHC, and education (Table 6).

**Table 6:** Working Alliance at T12. General Linear Model: ANOVA controlling for possible confounding variables.

	*F*	**Sig**.
Intercept	41.31	.000
Education	.24	.628
Time from the first contact with MHS	4.83	.037*
Group (STM or SB-APP)	6.99	.013**
Sex	.13	.718
Group * Sex	.01	.992

STM = Supervised Team Management group; SB-APP = Sequential Brief Adlerian Psychodynamic Psychotherapy group.

Dependent variable: WAI-S Total Score. * = inverse correlation between WAI-S score and time since the first contact with MHS; ** = the WAI-S score is higher in the SB-APP group than in the STM group (see text).

*df *= 1

All *post-hoc *comparisons were made with Bonferroni's test (corrected for number of comparisons). Due to the explorative nature of the study we considered significant an *alpha *level of .05 (two-tailed). According to an intention-to-treat analysis, only those individuals lost to follow-up were not included in data analysis (one patient in each group).

## Results

### Sample Description

Seventeen individuals (48.6%) were female, and 18 (51.4%) were male. Their mean age was 39.5 (SD = 9.4; range 24-57), mean CGI was 4.1 (SD = .9; range 2-6), and mean GAF was 58.7 (SD = 9.3; range 30-75). Only 13 participants (37.1%) did not show a comorbid Axis I diagnosis (DSM-IV-TR).

### Dropout rate

Few participants dropped out during this study: one member of the STM branch left between T3 and T6 (no more contact with the MHS), and another one left the SB-APP group between T3 and T6 (this patient maintained contact with the MHS). The first patient was in contrast with staff members on an impulsivity base, and the second dropped out because of the initial requests of changes during SB-APP.

Two patients of STM group refused to complete the questionnaires between T6 and T12, and one in SB-APP group refused to complete the questionnaires between T6 and T12 but they were included in the statistical analysis using hetero-administered measures.

### Comparison at T0 between the two treatment groups

The two treatment groups did not differ significantly after randomization (Table 1). The distribution of Axis I disorders in the two groups did not significantly differ (*chi*-square: 2.402, *p *< .791): Eating Disorder Not Otherwise Specified (1 patient in STM group vs 1 in he SB-APP group), General Anxiety Disorder (2 vs 2), Unipolar Mood Disorder (3 vs 2), Dysthymia (4 vs 6), Obsessive-Compulsive Disorder (0 vs 1), and no Axis I disorder (7 vs 6).

### Comparison of scores at T0, T3, T6, and T12

Tables 2 and 3 show the scores of self-administered questionnaires (SCL-90-R Total score, STAXI) and clinical rating scales (CGI, GAF, and CGI-M 9 items) at intake (T0), after three (T3), six (T6), and 12 months (T12). Independently of treatment group, patients were found to overall improve in terms of general symptoms and clinical severity (SCL-90-R; CGI; CGI-M 9 items), global functioning (GAF), and control of anger expression (STAXI) at T6 and T12. SB-APP treatment seemed more effective than STM on four CGI-M items at T6 and T12 (Table 3): disturbed relationships (*p *< .040), impulsivity (*p *< .025), self-damaging behaviors (*p *< .019), and chronic feelings of emptiness (*p *< .009).

### Comparison of scores at T12, T18, and T24

Table 4 shows the scores of clinical rating scales (CGI, GAF, CGI-M 9 items) at 12 (T12), 18 (T18), and 24 months (T24). Not significant changes emerged in both treatment groups during the year after conclusion of psychotherapy.

### Clinical status before and after STM and SB-APP treatment

Using a GLM for repeated measures, the number of days of hospitalization and self-harming episodes during the prior year (T0) was compared with the number of days of hospitalization and self-harming episodes during the year of treatment (T12) and after conclusion of STM or SB-APP intervention (T24).

No significant difference was found between T0 and T12 in each measure.

We found a significant reduction in the number of days of hospitalization during the year after conclusion of treatment (T24; STM: 4.8 ± 9.8; SB-APP: 8.2 ± 19.1) compared with the year before the study (T0; STM: 21.1 ± 58.1; SB-APP: 31.2 ± 66.5), independently of the delivered treatment (*F *for time = 4.937; *p *< .033; *F *for time*group = .142; *p *< .709).

An overall reduction of self-harming episodes was recorded for both groups during the year before the time-limited psychological treatments (T0; STM: 2.3 ± 3.1; SB-APP: 3.1 ± 2.5) and the year following their conclusion (T24; STM: .4 ± .8; SB-APP: .3 ± .6), irrespective of treatment group (*F *for time = 33.370; *p *< .000; *F *for time*group = 1.357; *p *< .252).

### Heavy users and Global Functioning during the Follow-up year

The number of performed sessions at T12 was planned to be the same (40 vs 40 weekly sessions). Supportive setting was maintained until T24 and so it did not significantly differ between STM (n = 85.4 ± 12.2) and SB-APP (n = 80.7 ± 6.3) groups (*t *= 1.403; *p *< .171).

Only 23.5% (n = 8/34) of the sample met the criteria for MHS heavy use at T24 (*chi*-square = 38.919; *p *< .001).

The proportion of individuals in the two groups requiring more than six emergency interventions, first criterion for heavy use of MHS, did not differ at T24 (n = 5/16 in the STM group and n = 3/18 in the SB-APP group; *chi*-square = .443; *p *< .311).

The proportion of patients in the two groups with GAF scores of at least 60 at T24 did not differ (n = 10/16 in the STM group, n = 14/18 in the SB-APP group; *chi*-square = .457; *p *< .275).

Patients with lower GAF scores at T24 were also more likely to remain heavy users at this time (n = 5/8 heavy users had GAF < 60 at T24; *F = *5.517; *p *< .031). Thus, individuals without sufficient improvement in symptoms and social functioning after two years of treatment remained heavy users of MHS.

### Working Alliance

The WAI-S total score at T12 using GLM ANOVA (Table 5) was found to be related to two independent factors: time from the first contact with MHS (TIME; *r *= -.329; *p *< .048) and treatment group (SB-APP vs. STM) with total scores on working alliance higher in SB-APP group at *post-hoc *analysis (STM: 46.7 ± 8.8 and SB-APP: 53.2 ± 6.3; *t *= -2.426; *p *< .021; Table 5).

## Discussion

Three main results emerged from this study: 1) the branch of the study including specific MHS team supervision in addition to treatment-as-usual (STM) showed an improvement in the symptoms and functioning compared to baseline, even though a structured psychotherapy was not applied; 2) the improvement was found to be stable over time; 3) a time-limited psychodynamic psychotherapy focused on patients' level of personality functioning (SB-APP) was more effective than STM with respect to some core psychopathological characteristics of BPD (disturbed relationships, impulsivity, suicidality/self-damaging behaviors, and chronic feelings of emptiness) and working alliance.

With regard to the first point, both patients and raters reported that one year of treatment as usual, comprehending specific MHS team training and supervision, improved in both psychopathological expression and substantial reduction of heavy use of MHS. These data provide support to design a randomized controlled trial to demonstrate that the addition of team training may have a role in obtaining these effect. The whole group of patients reported a significant improvement in general psychopathology and in anger control during treatment and at the first-year follow-up. STM effectiveness is even more extensively confirmed by hetero-administered scales, showing a significant improvement in all CGI scores.

These results are in line with previous studies and are interesting because a better management of anger is an important target when treating BPD patients [27]. This may be consequent to a greater consistence of MHS team management and/or to a reduction of counter-transferal reactions by therapeutic team which could elicit BPD patients' anger [28]. Indeed, anger reactions are often explosive and prolonged, constituting indeed major risks factors for suicidality [29].

An overall improvement in clinical severity (CGI), global functioning (GAF), and all nine psychopathological domains included in the diagnosis of BPD (CGI-M items 1-9) were reported by blind, trained researchers. The overall improvement in global functioning (GAF) after one year is particularly important because personality disturbances lead to social dysfunction to a greater extent than the majority of other pathologies and patients quality of life may remain poor even after well-designed therapeutic interventions (i.e. Cognitive Behavioral Therapy for Personality Disorders - CBT-PD) [30]. Besides, poor social adjustment is a risk factor for suicide attempts among patients with BPD [29]. Moreover, individuals who did not reach a sufficient GAF level remained heavy users of MHS at T24.

The improvement of anger control in our sample could have a role on reduction in self-damaging behaviors and hospitalization rates during the year after psychotherapeutic treatment. Further studies are necessary to confirm this causal hypothesis.

Finally, the significant improvement of the main outcome measure of this study, i.e. recurrence of high use of psychiatric service at the two-year follow-up, showed that the interventions were effective on this core problem afflicting both patients and service. These results are consistent with literature findings. Among medium-term interventions ranging from 12 to 18 months, one year of DBT was superior both to treatment-as-usual (TAU) and treatment by community experts with regard to self-harm, suicidality, hospitalization and psychiatric emergency visits reduction [9]. Also eighteen months of Mentalization Based Treatment (MBT) showed better results than TAU concerning a steeper decline of both self-reported and clinically significant problems, including suicide attempts and hospitalizations [31]. Similarly, Transference Focused Psychotherapy (TFP) [32] was more effective than treatment by experts in reducing suicidality and in inpatient psychiatric treatment, but not in self-harming behaviors. Finally, there is evidence of benefit from adding CBT-PD to TAU on suicidal attempts, state anxiety and dysfunctional beliefs [33]. Among brief treatments, a shorter form of DBT (DBT-B; 6 months of duration) lead to significant decreases in self-injure, suicide ideation and overall patients subjective distress, but not to a reduction of hospitalizations and emergency visits [34].

BPD patients are high users of MHS because they need several repeated treatments, including urgent interventions either in community settings or in emergency departments [2,9]. The reduction of dramatic occurrences and unscheduled interventions in both groups of patients underlines that a significant component of borderline malfunction can be reduced correcting therapeutic behaviors that worsen symptoms [28,35] by a specific MHS team training. Working with a precise theoretical framework and being supported by a specialist supervision which addresses relational dynamics of the therapeutic team is useful with BPD, a disorder which heavily challenges consistence and coherence of operators [5,12]. Data showed in this study strongly encourage MHS teams to adopt similar supervision procedures.

As regards the second finding of this study, our data analysis of the scores at the second-year follow-up did not show any significant change in psychopathology or clinical scores and no significant differences at the between-group analysis were found. Nevertheless, the evaluation of heavy use status underscores that at the two-year follow-up the majority of BPD patients are no more heavy MHS users. This sustains a stability of improvement one year after the beginning of the intervention. This stability has been supported by the persistence of the STM with team supervision in the follow-up period. Nevertheless, this suggests that: 1. improvement from the initial therapeutic approach (without team supervision) to the therapeutic approach with team supervision (STM) leads to an enduring better functioning of BPD patients; 2. STM with supervision alone is not able to significantly further improve patients' functioning after one year of treatment; 3. improvement of BPD functioning reached with a better clinical management is sufficient to significantly reduce the heavy use of the service at two-year follow-up; 4. more specific treatments (possibly SB-APP) are needed to further improve BPD psychopathology beyond the functioning level obtained in first year of both treatments. In fact, the high non-homogeneity of BDP diagnoses require tailored treatments based on specific clinical, psychopathological and functioning (PLF) characteristics [13].

These data have to be confirmed by longer follow-up periods. BPD patients treated with CBT-PD showed a stable improvement after 6 years [30] and patients treated with MBT after 8 years [36].

As concerns the third finding of this study, one of the major aims was to compare two different therapeutic techniques: STM with and without a structured cycle of SB-APP. Research supports that BPD treatment may benefit from a structured approach to psychotherapy [31], since patients are often chaotic in their lives and relationships and have a deficient psychic structure [8]. The effectiveness of well structured treatments might widely relate to common factors such as stable framework, active and empathic therapist, attention to countertransference and progressive patients' awareness connecting feelings, thoughts and behaviours [37-39] and not to specific techniques [8]. Nevertheless, focusing on specific psychopathological processes could add benefits to structured clinical support [40].

SB-APP is a time-limited psychopathology-based intensive-supportive psychotherapy derived from B-APP, a 15-session time-limited treatment that has been used with good results [19], either alone or in combination with medication, for treating outpatients with mood and anxiety disorders [40] or eating disorders [41], also in comorbidity with personality disorders. The SB-APP was conceived because of the BPD individuals relationship disturbances, fears of abandonment and severe difficulties with building a stable therapeutic alliance [20].

Several treatments [9,30-34,36] are useful to address specific disruptive behaviors of severe BPD, but are less effective in reducing heavy MHS use related to intolerance of aloneness and conflicts over dependency [10] or the tendency of "pushing the limits" in building therapeutic alliance which produce a high rate of MHS use and great problems in BPD management [11].

Since the same therapists of the team performed both kind of interventions with different patients SB-APP, superiority to STM was not related to therapists' subjective skills but possibly to the specific setting and technique of the structured treatment with respect to the unstructured psychological support. In order to treat acting out and impulsivity (CGI-M, item 4) and self-damaging behaviors (CGI-M, item 5), an accurate identification of patients' cognitive and emotional patterns and defense mechanisms [15] is required: this represents the SB-APP specific focus. Distorted relationships and acting out (CGI-M, item 2) could also benefit from a well structured treatment setting. Moreover, patients feelings of emptiness (CGI-M, item 7) are very persistent and have different psychopathological features during evolution of BPD [21]. Consequently, SB-APP therapists address patients emptiness with either promoting mentalization [42] (PFL 1) or decreasing splitting defenses (PFLs 2 and 3) and increasing tolerance for ambivalence (PFL 4).

Considering remaining CGI-M items: dysphoria and anxiety (CGI-M, item 6), rage (CGI-M, item 8), as well as paranoid ideation (CGI-M, item 9) are likely to be decreased by pharmacological therapy. Increasing better skills to cope novelty with risk, as in unstructured psychological support, and providing empathic validation and encouragement to elaboration, as in SB-APP, are likewise useful to reduce affective and cognitive symptoms. Furthermore, treatment of fear of abandon (CGI-M, item 1) might benefit from implicit aspects of therapeutic relationship

Patients' identity instability (CGI-M, item 3) may be reduced by identification with therapists, irrespectively from their technique [43].

The client-rated quality of therapeutic alliance was rated only at the end of follow-up because of the difficulties in alliance-building and early termination of treatment [44]. Structured psychotherapy was more effective in building a good and stable therapeutic relationship. In fact, individuals treated with SB-APP described their therapist as more empathic and confident and rated a better working alliance than did those in the STM group.

A detailed cost-effectiveness analysis considering the savings due to the reduction of high MHS use (repeated hospitalizations, unscheduled sessions, at home interventions, loss of working days, etc.) was not performed in the present study to support the opportunity of SB-APP. SB-APP costs are represented by specific training to therapists and by the need of performing 40 structured weekly sessions with the psychotherapists. In our study, the total number of T0-T12 sessions was the same in the two treatment branches. Nevertheless, at T12 and T24follow-up SB-APP allowed to avoid the weekly psychological support which was carried on in the STM group saving about 40 weekly sessions (about 4.000, 00 $/participant).

## Conclusions

The hypothesis raised by the present research is that specific team training supervision and support on BPD psychopathology improve the outcome of MHS heavy users with respect to team management. These data provide support to eminent authors [5,12] claiming that working with a precise theoretical framework and being supported by a specialist supervision which addresses relational dynamics of the therapeutic team is useful when treating BPD.

In addition, the present study demonstrates that SB-APP showed superiority with respect to STM in some core psychopathological dimensions evaluated with the CGI-M: disturbed relationships, impulsivity, self-damaging behaviors (including suicidality), and chronic feelings of emptiness.

SB-APP offers an adjunctive outcome in these areas since it is focused on different core psychopathological features of BPD [14,15] compared to STM: supportive interventions are focused on individuals' impairment, while SB-APP is mainly focused on personality functioning.

Moreover, SB-APP has been found significantly more effective in building the therapeutic alliance.

Not unlike DBT-B and CBT-PD [34,30], SB-APP is a shorter therapy compared to twelve or eighteen-month treatments with DBT, TFP and MBT: however, it seems to be promising in order to treat severe patients with BPD in a more rapid way and with stable effects.

Finally, also nonspecific agents of structured time-limited psychotherapy, such as a specific setting and a more significant therapeutic relationship may be responsible of improved outcome with respect to unstructured psychological support.

### Limitations and perspectives

Concerning SB-APP group, we expected that the higher improvement in CGI domains and global functioning - compared with STM - would have produced in this group a greater reduction or remission of symptoms in the year after the psychotherapy cycle. On the contrary, also in SB-APP group a significant improvement was not found after the first year of follow-up. This may derive from severe resistance to change which is typical of personality disorders in general and of BPD patients in particular. This provides support to subsequent psychotherapy cycles to produce a progression in BDP disorder itself which goes beyond the mere overcoming of heavy MHS use.

Although these results are consistent with those of other authors [45], we should be cautious about their relevance due to the complexity of treatments and the need for further investigation. Larger samples are needed to confirm these preliminary results in real MHS clinical practice. Cost-effectiveness analyses are required to confirm effectiveness of present results [44,46]. Follow-up controlled studies are warranted to prove higher benefits of a structured psychotherapeutic treatment as SB-SAPP and its cost-effectiveness with respect to STM with supervision on the long-term period.

## List of abbreviations

B-APP: Brief Adlerian Psychodynamic Psychotherapy; BPD: Borderline Personality Disorder; CBT-PD: Cognitive Behavioral Therapy for Personality Disorders; CGI: Clinical Global Impression; CGI-M: CGI-modified for BPD; DBT: Dialectical Behavioral Therapy; DBT-B: Brief Dialectical Behavioral Therapy; EP-AX: Encouraging Position Axis; GAF: Global Assessment of Functioning; GLM - ANOVA: General Linear Model ANOVA; IS-AX: Intensive Supportive Axis; MBT: Mentalization Based Treatment; MC-AX: Mutative Conservative Axis; MHS: Mental Health Services; PFL: Personality Functioning Levels; SB-APP: Sequential Brief Adlerian Psychodynamic Psychotherapy; SCL-90-R: Symptom Checklist-90 Revised; STAXI: State-Trait Anger Expression Inventory; STM: Supervised Team Management; T: Time Point; TAU: Treatment-as-usual; TCI: Temperament and Character Inventory; TFP: Transference Focused Psychotherapy; TI-AX: Technical Instruments Axis; WAI-S: Working Alliance Inventory-Short Form, client version

## Competing interests

The authors declare that they have no competing interests.

## Authors' contributions

FA, AF and AP participated in the design of the study, performed statistical analysis and drafted the manuscript. EC, BR, BS, SIF recruited and assessed the participants, performed SB-APP and participated to the drafting of the manuscript. GAD and SEF participated in the design of the study and in the drafting of the manuscript. All authors read and approved the final manuscript.

## Author's information

All authors are psychodynamic psychotherapists. They work in public structures for health care and in University and/or Private Research Fundations for research in personality disorders. They share a psychodynamic training in Adlerian Psychotherapy. They are characterized by a deep experience and passion in personality assessment and psychotherapy of personality disorders. AF and SEF are also experienced Adlerian training analysts and eminent members of the International Association of Individual Psychology (IAIP). FA is member of the International Society for the Study of Personality Disorders (ISSPD).

### Financial Disclosure

No funding was received by any author to perform the present research which was introduced within the ordinary activities of the Chivasso MHS therapeutic team. The author SEF receiver funding from Bank Fundations San Paolo di Torino and Cassa di Risparmio di Torino (2001-2010), FA received funding for the National Eating Disorders Association (2005) for researches on eating disorders, AF received funding from Regione Piemonte (2010) for a research on self-harming in adolescents.

## Pre-publication history

The pre-publication history for this paper can be accessed here:

http://www.biomedcentral.com/1471-244X/11/181/prepub
